# Transcriptome analysis of different developmental stages of amphioxus reveals dynamic changes of distinct classes of genes during development

**DOI:** 10.1038/srep23195

**Published:** 2016-03-16

**Authors:** Kevin Yi Yang, Yuan Chen, Zuming Zhang, Patrick Kwok-Shing Ng, Wayne Junwei Zhou, Yinfeng Zhang, Minghua Liu, Junyuan Chen, Bingyu Mao, Stephen Kwok-Wing Tsui

**Affiliations:** 1 School of Biomedical Sciences, The Chinese University of Hong Kong, Hong Kong SAR, China; 2 Hong Kong Bioinformatics Centre, The Chinese University of Hong Kong, Hong Kong SAR, China; 3 Division of Infectious Diseases, Duke University Medical Center, Durham, North Carolina, USA; 4 State Key Laboratory of Genetic Resources and Evolution, Kunming Institute of Zoology, Chinese Academy of Sciences, Kunming, China; 5 Nanjing Institute of Paleontology and Geology, Chinese Academy of Sciences, Nanjing, China

## Abstract

Vertebrates diverged from other chordates approximately 500 million years ago and have adopted several modifications of developmental processes. Amphioxus is widely used in evolutionary developmental biology research, such as on the basic patterning mechanisms involved in the chordate body plan and the origin of vertebrates. The fast development of next-generation sequencing has advanced knowledge of the genomic organization of amphioxus; however, many aspects of gene regulation during amphioxus development have not been fully characterized. In this study, we applied high-throughput sequencing on the transcriptomes of 13 developmental stages of Chinese amphioxus to gain a comprehensive understanding of transcriptional processes occurring from the fertilized egg to the adult stage. The expression levels of 3,423 genes were significantly changed (FDR ≤ 0.01). All of these genes were included in a clustering analysis, and enrichment of biological functions associated with these clusters was determined. Significant changes were observed in several important processes, including the down-regulation of the cell cycle and the up-regulation of translation. These results should build a foundation for identifying developmentally important genes, especially those regulatory factors involved in amphioxus development, and advance understanding of the developmental dynamics in vertebrates.

Amphioxus is widely used as a model system for studying evolutionary developmental biology (evo-devo). It is a marine invertebrate that belongs to the subphylum Cephalochordata of the phylum Chordata. Fossil records and molecular phylogenetic analysis have positioned Cephalochordata as the close living invertebrate relative of the vertebrates^1^,^2^,^3^. Studies of amphioxus are usually carried out using the species *Branchiostoma* (*B*.) *floridae*, *B*. *belcheri* and *B*. *lanceolatum*. Due to the important role of amphioxus in evo-devo, the developmental processes of amphioxus have been fully characterized^4^. It is also notable that amphioxus displays anatomical and morphological characteristics that are intermediate between those of invertebrates and vertebrates. Thus, a more comprehensive analysis of the gene regulation control in amphioxus development would be helpful to improve understanding of the possible interface between non-chordates and chordates.

Through next-generation sequencing, the draft genomes of *B. floridae* and *B. belcheri* have been assembled^5^,^6^, resulting in an important resource for studying the molecular mechanism of amphioxus development. Apart from this genome sequencing, efforts to sequence expressed sequence tags (ESTs) have been carried out^7^, and ESTs of approximately 140,000 cDNA clones from five developmental stages (unfertilized egg, gastrula, neurula, 36-h larva and adult) of *B. floridae* have been generated. However, the functions of those sequences have not been categorized. Moreover, some cDNA clones might not be full-length, and some transcripts have not been identified, owing to their low expression levels. Although the transcriptome of *B. belcheri* has also been sequenced and used for annotation of the draft genome^6^, the regulation of genes that are involved in development remains elusive. Recently, the transcriptome of *Asymmetron lucayanum*, a cephalochordate distantly related to *Branchiostoma*, has also been sequenced^8^. Although the RNA libraries for transcriptome analysis were prepared from different developmental stages, the main focus of this study was molecular evolution instead of amphioxus development.

To gain a better understanding of the transcriptional processes occurring in the amphioxus development, in this study we sequenced the transcriptome of amphioxus across various developmental stages. Then, instead of using standard RNA Sequencing (RNA-Seq) approaches, we performed Digital Gene Expression Sequencing (DGE-Seq) on the transcriptome of each stage. Based on the expression patterns, differentially expressed transcripts were identified and classified into clusters. Finally, functional categories of each cluster were further characterized, providing a framework for the understanding of the developmental dynamics in amphioxus.

## Results

### Transcriptome Sequencing and *De novo* Assembly

**Figure 1:**
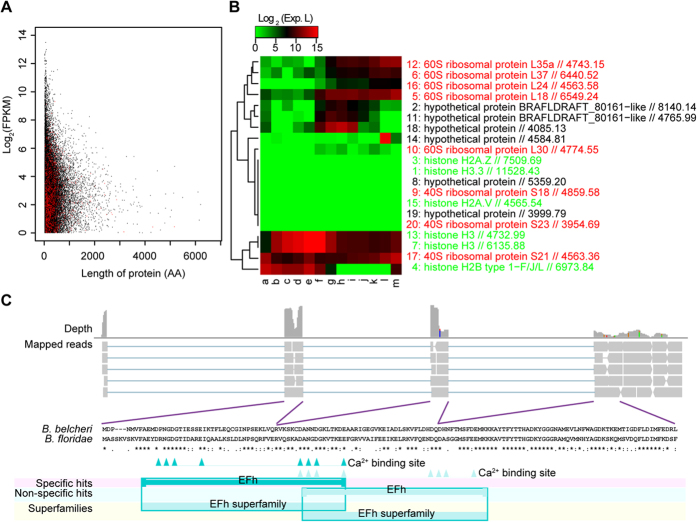
Overview of the transcriptome of amphioxus. (**A**) Distribution of the abundance of transcripts and the length of encoded proteins. The abundance of transcripts was calculated as FPKM with un-normalized RNA-Seq data. Transcripts that code for transcription factors are highlighted in red. (**B**) Heatmap showing expression patterns of top 20 highly expressed transcripts. Gene names are coloured according to their functions: red, ribosomal proteins; green, histone; black, hypothetical proteins. Values after gene names indicate the FPKM, which was estimated by Tophat-Cufflinks with un-normalized RNA-Seq data. Because they lacked a restriction site for the restriction enzyme *NlaIII*, the expression levels of several transcripts were not detectable across all stages by using DGE-Seq. a-m represent different developmental stages: a. egg, b. 2 cells, c. 16 cells, d. 64 cells, e. 128–256 cells, f. gastrula, g. neurula, h. hatch, i. 12 hours after hatching, j. 24 hours, k. 48 hours, l. male adult and m. female adult. (**C**) Features of a potential novel protein encoded by the transcript with highest expression level. Upper panel: alignment of RNA-Seq data on the amphioxus genome. Middle panel: pairwise alignment results of this protein with its counterpart in *B. floridae*. Lower panel: Domain structure of this protein.

To obtain a genome-wide gene expression profile for amphioxus development, we isolated total RNAs of 13 different developmental stages of *B. belcheri* from fertilized egg to adult (Materials and Methods). Two sequencing libraries from the pooled RNAs of all stages were constructed for sequencing with an Illumina Solexa sequencing system. At the same time, one adult library was prepared for sequencing with a Roche 454 sequencing system. In total, we obtained 97 million reads with a throughput of 8.2 Gb (Supplementary Table S1). In addition, RNA-Seq data from a previous study^6^ were downloaded from NCBI. *De novo* assembly of each library was performed with Trinity, and the protein-coding transcripts were further merged together to construct a final transcript set by using the Cluster Database at High Identity with Tolerance (CD-HIT) (Supplementary Table S2), whose completeness was assessed with the Basic Local Alignment Search Tool (BLAST) and Core Eukaryotic Genes Mapping Approach (CEGMA) (Supplementary Table S3). Based on the high percentage of protein homologues and core eukaryotic genes obtained, the transcript set of *B. belcheri* was nearly complete and therefore was suitable for further analysis. Using the non-normalized RNA-Seq data, we were able to estimate the abundance of each transcript. Among these transcripts, 1,948 were predicted to be transcription factors, most of which were expressed at a low level (Fig. 1A). Of the top 20 highly expressed transcripts, most encoded members of the histone family and the ribosomal protein family. However, the expression levels of these transcripts changed dynamically during development (Fig. 1B). Intriguingly that six highly expressed transcripts did not have any homologues in the non-redundant protein databases of NCBI. One of them had the second highest expression level of all transcripts. Interestingly, it was highly up-regulated during the neurula and hatch stages. Further analysis of this transcript suggested that it encodes a protein similar to the hypothetical protein BRAFLDRAFT_80161 (59% identity), and both of them contain an EF-hand calcium binding motif (Fig. 1C). Given its low identity (~30%) with its counterpart in other species, this protein could be a potential calcium-binding protein or calmodulin, which has been reported to participate in calcium signalling pathways related to neuronal development^9^,^10^. The expression pattern of this transcript also supports its potential role in this developmental stage.

### Dynamic Changes in Differentially Expressed Genes during Development

**Figure 2:**
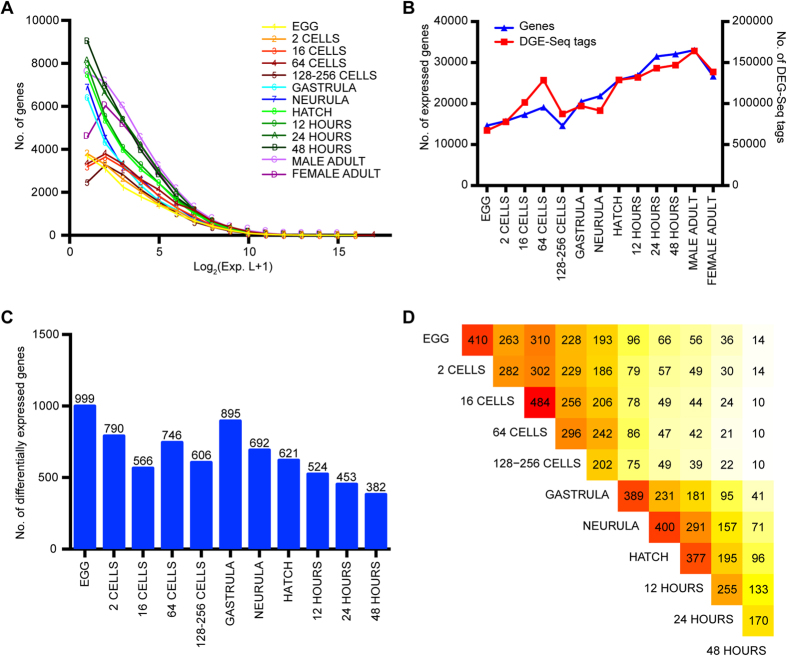
Expression profiles of transcripts across different developmental stages in amphioxus. (**A**) Distribution of expression levels (Exp. L.) across 13 developmental stages. The transformed expression values of transcripts at each stage were binned with interval size 1, and the number of genes falling within each bin was calculated. (**B**) Number of expressed genes and DEG-Seq tags in each developmental stage. Expressed genes were defined as those with log_2_(Exp. L.+1) > 0. (**C**) Number of differentially expressed transcripts at each developmental stage. Transcripts that were highly expressed in non-adult stages but down-regulated in the adult stage were calculated, with FDR ≤ 0.01. (**D**) Overlap of differentially expressed transcripts in each pair of developmental stages. The number of overlapped transcripts is represented by the colours.

To obtain a genome-wide gene expression profile during amphioxus development, we constructed 13 DGE-Seq libraries from different developmental stages of *B. belcheri*, including fertilized egg, 2-cell, 16-cell, 64-cell, 128–256-cell, gastrula, neurula, hatch, 12 hours after hatching, 24 hours after hatching, 48 hours after hatching, male adult and female adult stages (Supplementary Table S4). The expression levels of protein-coding transcripts were estimated with DGE-EM using DGE-Seq data. The distribution of gene expression levels in each stage was then examined to obtain an overview of the transcriptome of amphioxus during development (Fig. 2A). The difference in distribution between cleavage stages (from egg to 128–256 cell stage) and the following stages was mainly in weakly expressed transcripts, which contained approximately 40% of transcription factors (TFs) expressed in various developmental stages. During the cleavage stages, these weakly expressed maternal transcripts disappeared gradually as the developmental processes proceeded. At the 64-cell stage, the zygotic genome was activated, and increasing zygotic transcripts were expressed, resulting in an increase in expressed genes, so that increasing DGE-Seq tags were detected during this developmental stage (Fig. 2B).

To determine the dynamic changes in genes during development, especially those transcripts that were highly expressed in non-adult stages but down-regulated in the adult stage, we compared the expression levels of all transcripts in the male adult stage with those in other stages (Supplementary Fig. S1). We found that the Pearson correlation coefficient increased during amphioxus development (Supplementary Fig. S1). With a false discovery rate (FDR) ≤ 0.01, a total of 3,423 transcripts were considered to be stage-specific highly expressed transcripts (Supplementary Dataset S1). The highly expressed transcripts detected in each stage had a similar distribution to the distribution of Pearson correlation coefficient, except at the gastrula stage (Fig. 2C). The number of highly expressed transcripts increased at the 64-cell stage, which is consistent with the transcriptional activation of the zygotic genome during this period. Pairwise comparison of differentially expressed transcripts in each stage with that of all other stages suggested that the overlap of transcripts with adjacent stages was higher than the overlap with other stages (Fig. 2D).

**Figure 3:**
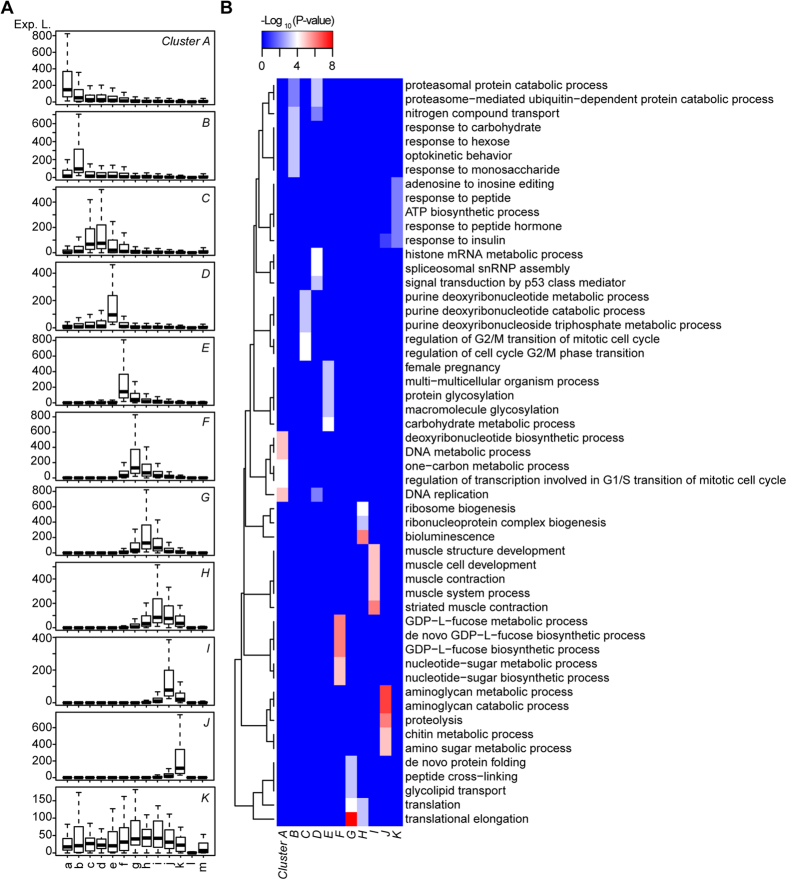
Expression patterns and GO enrichment of 11 clusters of differentially expressed transcripts. (**A**) Expression pattern for each cluster of non-adult-stage-specific differentially expressed transcripts. The expression level of each transcript in each cluster is measured in terms of expression values that were estimated by DGE-EM. a-m represent different developmental stages: a. egg, b. 2 cells, c. 16 cells, d. 64 cells, e. 128–256 cells, f. gastrula, g. neurula, h. hatch, i. 12 hours after hatching, j. 24 hours, k. 48 hours, l. male adult and m. female adult. (**B**) Heatmap plot of GO function enrichment. The heatmap plot is based on the top 5 GO terms of each cluster, which were enriched by using the top 100 differentially expressed transcripts of each cluster. The colours correspond to the significance (−log_10_(P-value)) of the enrichment in each stage.

When all 3,423 stage-specific highly expressed transcripts were included in a clustering analysis by using WeiGhted Correlation Network Analysis (WGCNA), 11 clusters with different expression patterns were generated (Fig. 3A). Except for cluster K, in which transcripts were expressed similarly during most of the developmental stages, other clusters were considered to be stage-specific according to their expression patterns. To determine the biological significance of these clusters, the enrichment of Gene Ontology (GO) terms in each cluster was determined using topGO (Supplementary Fig. S2). The top 100 most differentially expressed transcripts of each stage-specific cluster were also used for GO enrichment analysis (Fig. 3B and Supplementary Dataset S2). The results indicated a shift in the molecular functions of expressed transcripts during development.

### Transcripts Involved in Cell Cycle are Over-represented during Cleavage Stages

**Figure 4:**
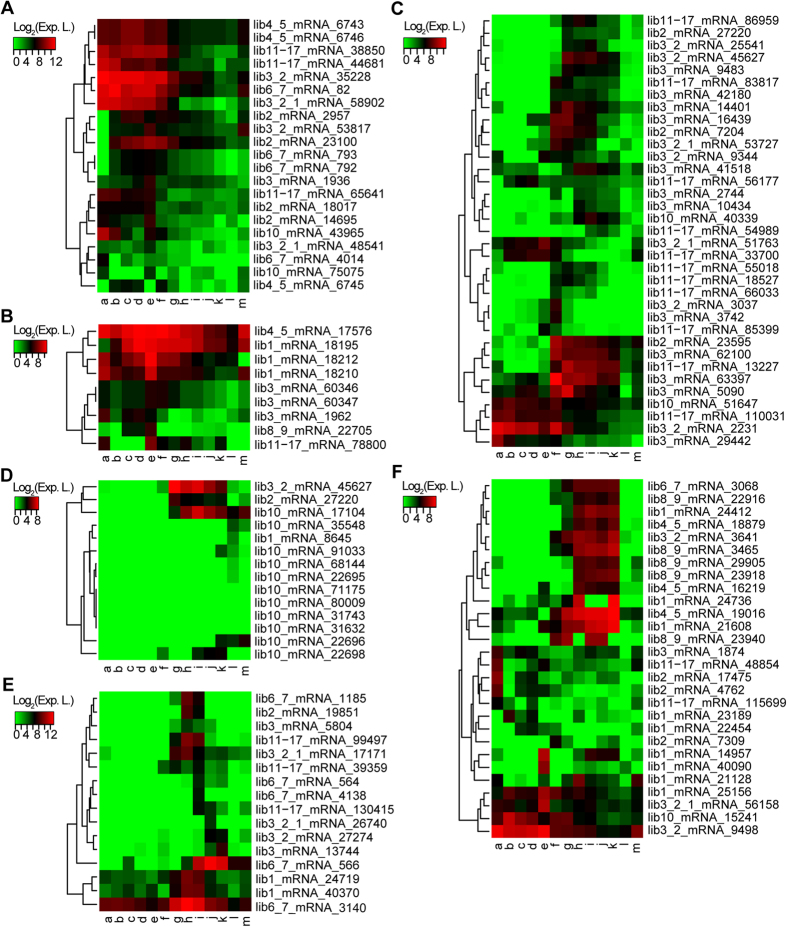
The distinct expression patterns of different groups of transcripts. (**A**) Heatmap of cyclin-related transcripts. (**B**) Heatmap of small nuclear ribonucleic proteins. (**C**) Heatmap of homeodomain-containing proteins. (**D**) Heatmap of Hox1-Hox14. (**E**) Heatmap of actin-related transcripts. (**F**) Heatmap of ribosomal proteins. The colours indicate the log_2_-transformed expression values, which represent the expression level of a transcript at each stage. a-m represent different developmental stages: a. egg, b. 2 cells, c. 16 cells, d. 64 cells, e. 128–256 cells, f. gastrula, g. neurula, h. hatch, i. 12 hours after hatching, j. 24 hours, k. 48 hours, l. male adult and m. female adult.

Before the activation of the zygotic genome, the major process of embryonic development is cleavage. Based on the GO enrichment analysis, a large number of differentially expressed transcripts in cluster A participated in small-molecule metabolic processes (Supplementary Fig. S2), which produce substances for other processes, such as the cell cycle. To better capture the nature of this cluster, the top 100 genes with the lowest FDRs were selected for functional annotation with topGO. As expected, significant enrichment of GO processes, including the cell cycle and DNA replication, as well as deoxyribonucleotide biosynthetic processes, were found in this subset of highly expressed transcripts (Fig. 3B and Supplementary Dataset S2). Similar results were found in the next three clusters (cluster B–D). Among the highly expressed transcripts in the first four clusters, 22 transcripts were related to cyclins, which control the cell cycle by interacting with cyclin-dependent kinases. Consistently with the reduction in cell division rate during development, most of the cyclin-related transcripts were gradually down-regulated from the egg to the adult stage (Fig. 4A).

At the end of cleavage stages, another group of GO biological processes were significantly enriched, including transcription-related processes, in particular the mRNA splicing process. The expression of this group of genes was detected before the 128–256-cell stage, suggesting that the zygotic genome had been activated before this period. A set of small nuclear ribonucleoproteins (snRNPs) and their associated proteins, which are essential to the removal of introns from pre-mRNAs, were found in these four clusters. The expression patterns of these transcripts further confirmed the activation of zygotic transcription (Fig. 4B).

### Homeodomain-containing Proteins are Activated during Gastrulation

TFs are important regulators of different cellular processes in development, and changes in the expression of transcription factors allows for transcription factor-mediated gene activation. Members of the homeodomain-containing gene family are essential regulators controlling the development of body patterning of various species. In our datasets, the expression of 35 homeodomain-encoding genes significantly changed during amphioxus development (Fig. 4C). Among them, 11 genes were detected in cluster E, whose transcripts were highly expressed in the gastrulation stage. These included several well-known homeodomain-containing proteins, such as SIX4 (lib2_mRNA_23595), GSC (lib3_mRNA_16439, lib11-17_mRNA_66033), DIX1 (lib3_mRNA_62100), VENT1 (lib2_mRNA_7204, lib3_mRNA_42180), OTX (lib3_mRNA_63397) and OTP (lib11-17_mRNA_83817). In fact, the expression of *Gsc*, *Vent1*, *Dlx1* and *Otx* at the gastrula stage has been previously reported in amphioxus^11^,^12^,^13^,^14^,^15^. Among these homeodomain-encoding genes, *Otx* was the most highly expressed gene at the gastrula stage. OTP is a protein highly conserved during evolution and is expressed in the central nervous system both in mouse and Drosophila^16^. Unlike other homeodomain-encoding genes in cluster E, *Otp* was not detectable until the gastrula stage (Fig. 4C and Supplementary Dataset S1), as in Drosophila^16^. Last, a novel homeodomain-containing protein (lib3_2_1_mRNA_53727) was up-regulated in the gastrulation stage. Interestingly, this protein was highly conserved in amphioxus (81% identity with the hypothetical protein BRAFLDRAFT_128160) but could not be found in any other species (<40% identities), suggesting that this could be a species-specific gene. This gene appeared to be down-regulated soon after gastrulation, indicating that it may be a gastrulation-specific gene.

### Distinct Processes and Transcription Factors are Involved in Neurulation

After gastrulation, organogenesis begins. Its first stage is neurulation, in which the nervous system is formed. Our results showed that 286 transcripts were highly expressed in the neurula stage. In the GO enrichment analysis, GDP-L-fucose-related processes were identified (Fig. 3B and Supplementary Dataset S2). GDP-L-fucose, the product of these processes, functions as a fucose donor in the synthesis of the fucosylated glycans, which guide the migration of vagus motor neuron progenitors during the development of hindbrain in zebrafish^17^. Within the top list of differentially expressed genes, a number of proteins related to the cytoskeleton were found, including keratin type I (lib11-17_mRNA_13337) and calmodulin (lib1_mRNA_32780). The expression patterns of these genes were similar to those previously reported in amphioxus^18^,^19^. Several transcription factors were also included in this cluster. Notably, *Cf1a* (lib3_mRNA_5090), *Coe3* (lib10_mRNA_84913, lib3_2_mRNA_29203 and lib3_2_mRNA_29213), *Cdx-4* (lib11-17_mRNA_13227) and *Sp5* (lib3_2_mRNA_56454) were expressed at relatively high levels (Supplementary Dataset S1). In amphioxus, COE family genes are expressed in the central nervous system and on the flanks of neurula stage embryos^20^, and CDX family genes are detected dorsally in the neuroectoderm at the beginning of neurulation^21^. However, the participation of CF1A and SP5 at the neurula stage in amphioxus has not been reported before. In Drosophila, the expression patterns of *Cf1a* suggested that it may play multiple roles in specific ectodermal cells and the embryonic nervous system^22^. Studies on the Xenopus SP5 have also implied that it functions as a critical early factor regulating neural crest induction^23^, although amphioxus lacks a neural crest^24^. HOX1 (lib3_2_mRNA_45627), another important homeodomain-containing protein, was also found in this cluster. Other homeobox genes in amphioxus (HOX2 - HOX15) were found in the final transcript set; however, they were not considered to be differentially expressed genes because of their low expression level during amphioxus development (Fig. 4D).

### Actins and Ribosomal Genes are the Major Differentially Expressed Genes After Hatching

After hatching, the embryo of amphioxus undergoes morphogenesis, organogenesis and then transformation into a larva. Cluster G-J contained genes that were involved in this process. GO terms such as translation, muscle system process and aminoglycan metabolic process were enriched (Fig. 3B and Supplementary Dataset S2). Among the 763 differentially expressed genes in these stages, 16 actin-related transcripts were identified. Although the expression patterns of these transcripts were quite different, most of them were activated at the hatch stage (Fig. 4E). In addition, 13 out of 28 ribosomal proteins that were differentially expressed were also found in these clusters. The activation of the ribosomal protein genes after hatching is in line with the need for the synthesis of large amounts of proteins at that stage (Fig. 4F).

## Discussion

Many modern developmental biology studies have focused on the genetic control and the expression of developmental genes that might participate in the processes of cell growth, differentiation and morphogenesis, which give rise to tissues, organs and the body plan. Recent advances in high-throughput sequencing technologies have had an immense impact on transcriptome analysis, which provides an opportunity to study the underlying mechanisms regulating developmental events in amphioxus. Here, we applied RNA sequencing technology to study the transcriptomes of Chinese amphioxus and generated comprehensive information about the dynamic changes in gene expression during the development of *B. belcheri*. By comparing gene expression profiles at different developmental stages, we provide a framework for the future investigation of developmental processes in amphioxus.

One of the important questions in evolutionary and developmental biology is how species can share similar developmental genetic toolkits but still generate diverse life forms^25^. It has been suggested that phenotype diversity derives from differences in where and when genes are expressed, rather than in the products that the genes encode. Owing to the rapid development of sequencing technologies, the components of development, which include TFs, cell adhesion proteins, cell surface receptor proteins, and secreted morphogens, in different species have become accessible. Among various model organisms, the zebrafish (*Danio rerio*) has contributed significantly to the understanding of vertebrate development. Recently, the dynamics and diversity of the transcriptome in the early stages of zebrafish embryogenesis have been well studied^26^,^27^,^28^,^29^. These resources make the zebrafish a good model for comparative transcriptomic analysis with amphioxus.

In general, the control mechanism of development of all animals passes through two stages. The first one is handled by maternally provided gene products, whereas the second one is conducted by those gene products synthesized from the zygotic genome^30^. This is called the maternal-to-zygotic transition (MZT) or the mid-blastula transition (MBT). Before the MZT, the fertilized eggs undergo the cleavage period, in which maternally stored transcripts direct early development and will be degraded either gradually or quickly later on. In both zebrafish and amphioxus, GO enrichment analysis of the differentially expressed transcripts in the first few stages shows that they are related to cell cycle processes. The degradation of maternal cell cycle transcripts results in the gradual increase of mitotic cycle length and permits the activation of zygotic genome, which is common in the animal kingdom^30^. During this period in zebrafish, most maternal transcripts undergo an increase in poly-A tail length, which leads to better efficiency of translation. Similar mechanisms might also occur in amphioxus, which may explain why increasing transcripts became detectable by DGE-Seq using oligo(dT) primers. Another mechanism that relates to the activation of the zygotic genome is DNA demethylation. One of the protein candidates that has a role as a pre-MZT transcriptional repressor is DNA methyltransferase 1 (encoded by *Dnmt1*). This protein directs terminal tissue differentiation in zebrafish, and knocking down *Dnmt1* causes an increase in embryo mortality^28^,^31^. In our study, a similar differential expression pattern of a *Dnmt1* homologue (lib3_2_1_mRNA_53077, Supplementary Dataset S1) was detected, with a decreasing trend of expression during the progression of development. This might reflect its function in suppression of transcription during pre-MZT.

The next event of the MZT is zygotic genome activation (ZGA). Different cohorts of genes are zygotically transcribed at discrete time points. During the blastula and gastrula stages of zebrafish development, zygotic transcripts encoding most TFs involved in differentiation, pattern formation and cell morphology, including ZIC3, SOX3, FOXA3 and FOXD3, can be grouped into the MBT clusters of genes, which are expressed at low levels at the pre-MBT stages and increase in abundance starting from the MBT^27^. Homologues of these transcription factors (Zic3: lib10_mRNA_32951, Sox3: lib3_mRNA_7212, Foxa3: lib_mRNA_45088, Foxd3: lib10_mRNA_64532) were found in the amphioxus transcriptome, and their expression patterns were similar to their counterparts in zebrafish. The other two proteins, Pou-domain class 5 transcription factor 1 (POU5F1) and TATA-binding protein (TBP), regulate some genes expressed at the ZGA^32^,^33^. Moreover, *Pou5f1* has been detected at the 1-cell stage and shows a large increase in the 50% epiboly stage (gastrula)^28^. However, the amphioxus homologue of *Pou5f1* (lib3_mRNA_57870) showed a different expression pattern in this study (Supplementary Fig. S3). In contrast, *Tbp*, which is important in transcription initiation, is present as a pre-MBT transcript in zebrafish, and its expression increases from the egg stage to a maximum at the mid-blastula stage, an expression pattern that is similar to that of the *Tbp* homologue (lib2_mRNA_26819) in amphioxus (Supplementary Fig. S3). It is important to note that the exact time of zygotic gene activation may not be inferred from our results because the number of transcripts may be low and therefore undetectable by RNA-Seq. However, the up-regulation of the *Tbp* homologue at the 16-cell stages supports the idea that the zygotic genome is already activated by this period in amphioxus.

In conclusion, our data illustrated the expression patterns of distinct classes of genes that may be involved in the development of amphioxus. Comparison of these expression patterns with other species, especially vertebrates, showed some common developmental features shared by the animal kingdom. More comprehensive studies on those differentially expressed genes will certainly enhance understanding of the developmental dynamics in amphioxus.

## Materials and Methods

### Sample Preparation, cDNA Library Construction and Sequencing

Chinese amphioxus, *B. belcheri*, were collected and reared in Beihai, Guangxi, China. The mRNAs from male adult amphioxus were extracted for Roche 454 sequencing (Lib-1). Two cDNA libraries were constructed from total RNAs of samples from different developmental stages, including fertilized egg, 2-cell, 16-cell, 64-cell, 128–256-cell, gastrula, neurula, hatch, 12 hours, 24 hours, 48 hours, male adult and female adult stages and were sequenced with Illumina Solexa paired-end sequencing (Lib-2 with normalization and Lib-3 without normalization). The RNA libraries from each stage were also sequenced through DGE-Seq by BGI as described in the Illumina DGE-Seq protocol. All sequencing data were submitted to the National Center for Biotechnology Information (NCBI) (BioProject: PRJNA310680).

### *De novo* Assembly and Reducing Redundancy of Assembled Transcripts

Additional RNA-Seq data from a previous study^6^ were downloaded from NCBI (SRX344152 to SRX344156). Adapters and low-quality bases were removed before assembling. *De novo* assembly of each data set was performed with Trinity^34^. Protein-coding transcripts were merged with CD-HIT^35^. The completeness of the transcript set was confirmed with BLAST and CEGMA^36^. The abundance of the final transcript set was estimated by mapping RNA-Seq data from Lib-3 with Bowtie^37^ and RSEM^38^.

### Detection of Differentially Expressed mRNA Transcripts in Different Developmental Stages

Clean reads from DGE-Seq from each developmental stage were mapped to the final transcript set with DGE-EM (v1.0.0)^39^, and the expression level of each transcript was estimated. EdgeR^40^ was used to detect the differentially expressed transcripts by comparing the male adult stage with other stages. Transcripts that were highly expressed in non-adult stages but weakly expressed in adult stages were considered as our targets.

### Construction of Co-expression Network (Clusters) of Differentially Expressed mRNA Transcripts and Gene Ontology Enrichment Analysis

WeiGhted Correlation Network Analysis (WGCNA) was used for identifying clusters of co-expressed transcripts. Gene Ontology enrichment of each cluster was performed by using topGO.

## Additional Information

**How to cite this article**: Yang, K. Y. *et al*. Transcriptome analysis of different developmental stages of amphioxus reveals dynamic changes of distinct classes of genes during development. *Sci. Rep*. **6**, 23195; doi: 10.1038/srep23195 (2016).

## Supplementary Material

Supplementary Information

Supplementary Dataset 1

Supplementary Dataset 2
