# Supplementary material for: Transcriptome analysis of different developmental stages of amphioxus reveals dynamic changes of distinct classes of genes during development
Source: Sci Rep. 2016 Mar 16;6:23195. doi: 10.1038/srep23195 (PMC4793263; doi:10.1038/srep23195)
Supplement: Supplementary Information [file srep23195-s1.pdf]

Supplementary Information

Transcriptome analysis of different developmental stages of amphioxus reveals dynamic changes of distinct classes of genes during development.

Kevin Yi Yang[1,2,+], Yuan Chen[3,+], Zuming Zhang[4], Patrick Kwok-Shing Ng[1], Wayne Junwei Zhou[1], Yinfeng Zhang[1], Minghua Liu[1,2], Junyuan Chen[5], Bingyu Mao[4,*], Stephen Kwok-Wing Tsui[1,2,*]

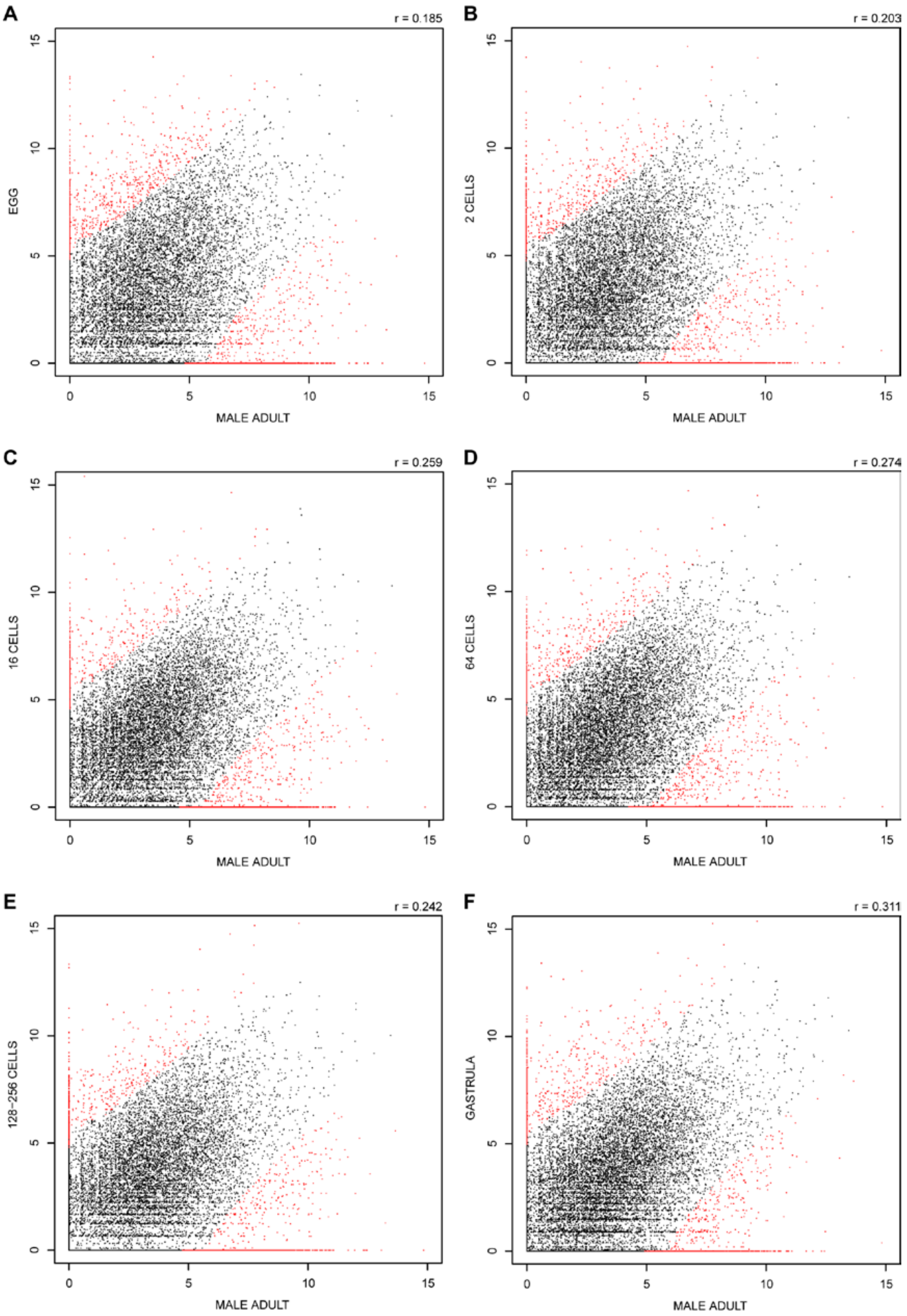
A
r = 0.185
EGG
MALE ADULT
B
r = 0.203
2 CELLS
MALE ADULT
C
r = 0.259
16 CELLS
MALE ADULT
D
r = 0.274
64 CELLS
MALE ADULT
E
r = 0.242
128-256 CELLS
MALE ADULT
F
r = 0.311
GASTRULA
MALE ADULT

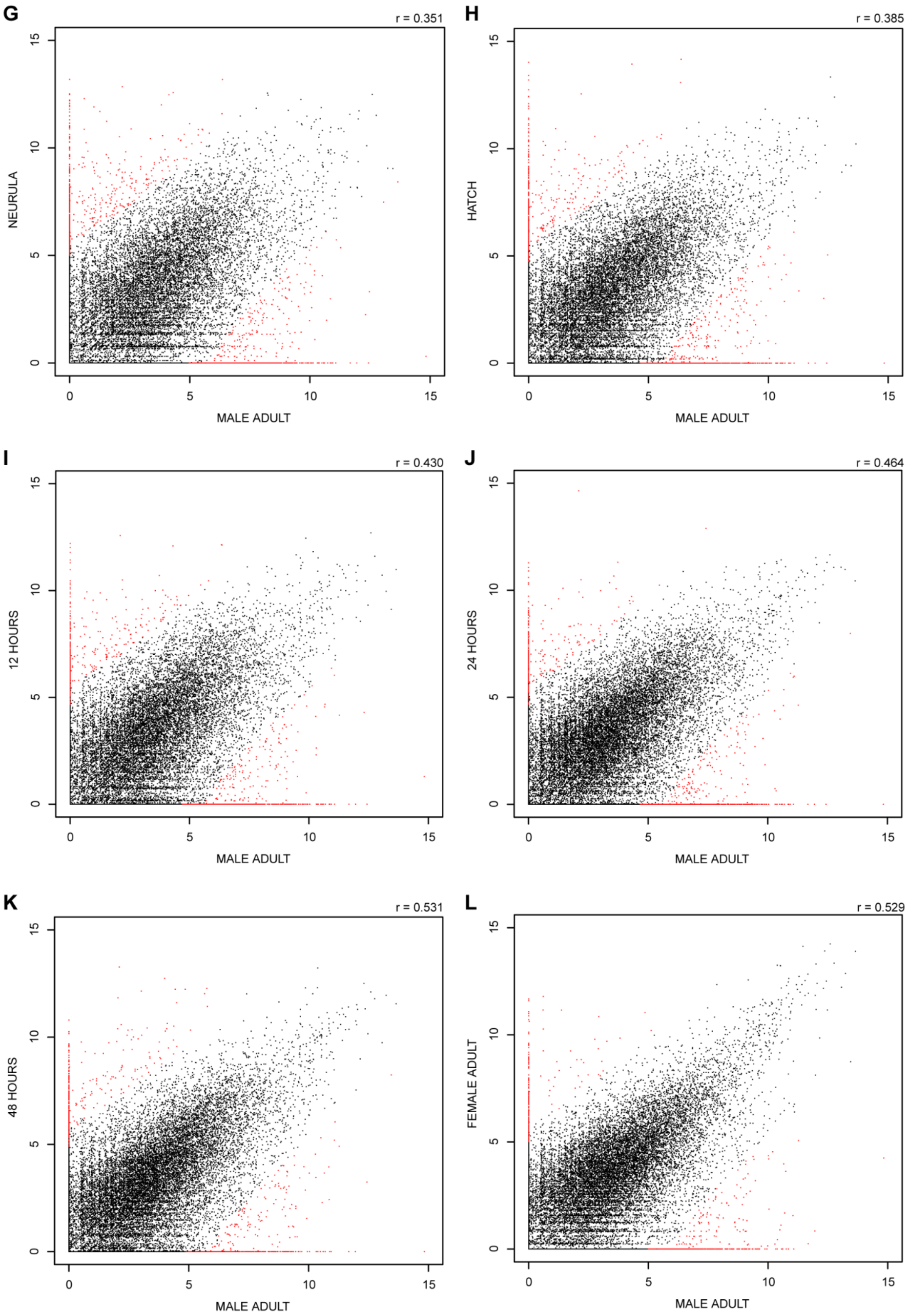
G
r = 0.351
NEURULA
MALE ADULT
H
r = 0.385
HATCH
MALE ADULT
I
r = 0.430
12 HOURS
MALE ADULT
J
r = 0.464
24 HOURS
MALE ADULT
K
r = 0.531
48 HOURS
MALE ADULT
L
r = 0.529
FEMALE ADULT
MALE ADULT
0
5
10
15

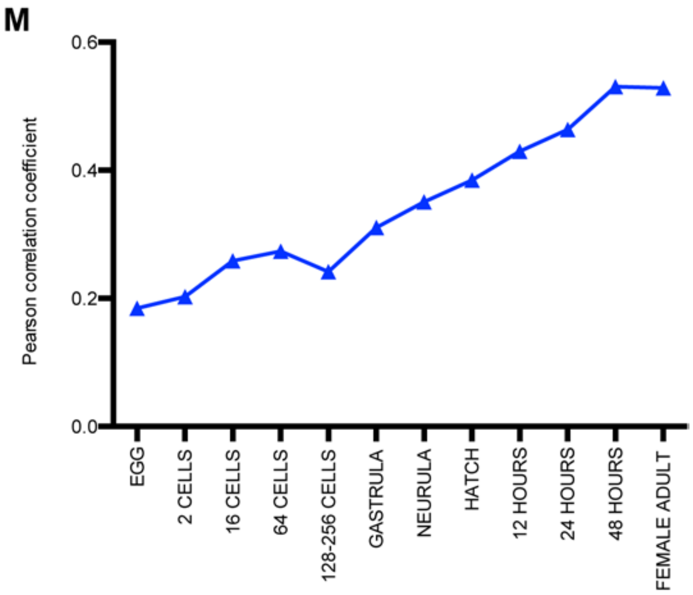

Supplementary Figure S1. Comparison of expression level of transcripts at each stage with male adult stage. (A-L) Log-log scale plot of expression value of each transcripts by male adult stage with other stages: (A) Egg; (B) 2 cells; (C) 16 cells; (D) 64 cells; (E) 128-256 cells; (F) Gastrula; (G) Neurula; (H) Hatch; (I) 12 hours after hatching; (J) 24 hours; (K) 48 hours; (L) Female adult. The logarithm is based on 2. Red plots indicate significant differentially expressed transcripts. (M) Distribution of Pearson correlation coefficient for each comparison.

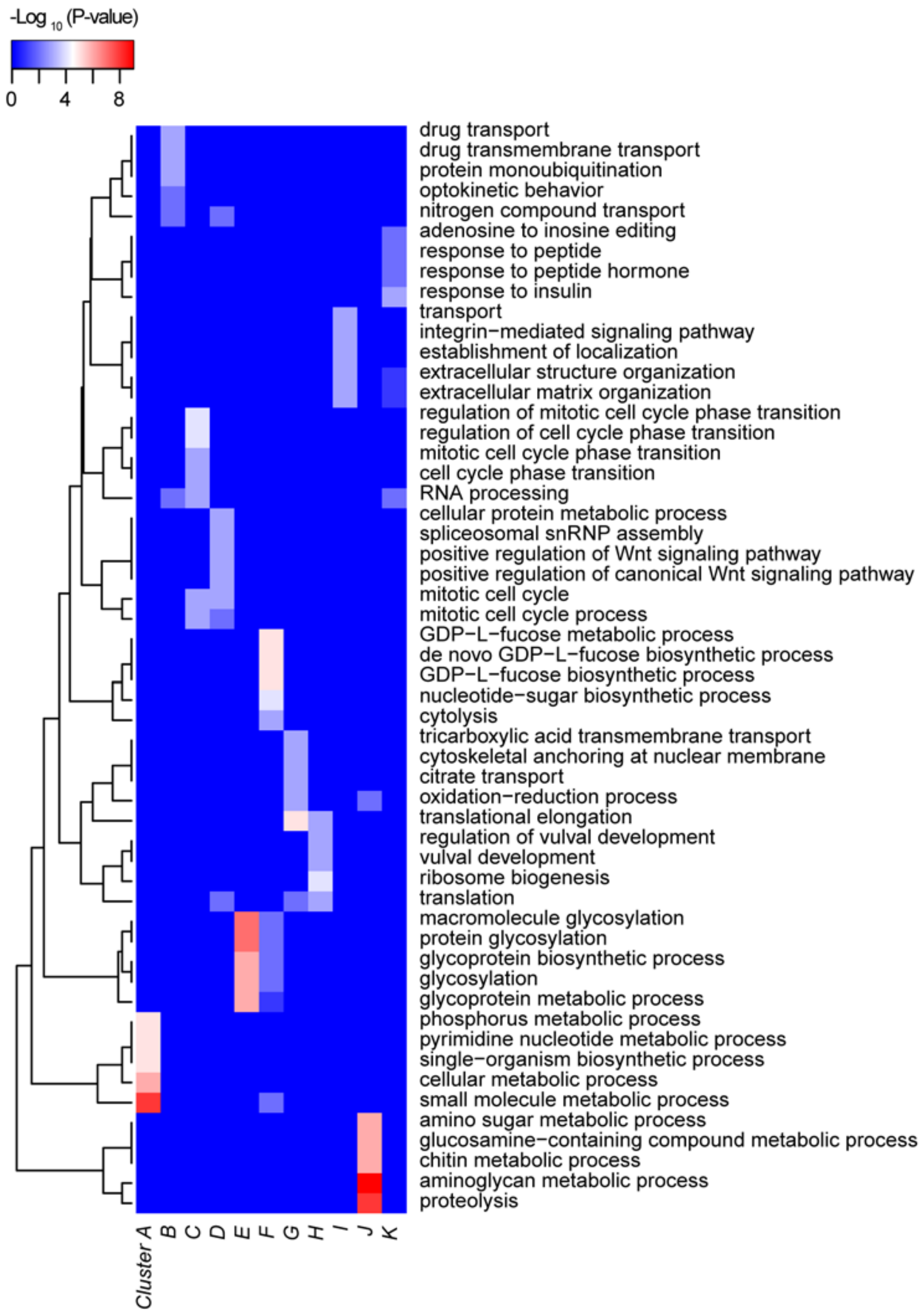

Supplementary Figure S2. GO enrichment of 11 clusters of all differentially expressed transcripts.

Heatmap plot of GO function enrichment. The heatmap plot is based on the top 5 GO terms of each cluster, which were enriched by using all differentially transcripts of each cluster. The colors correspond to the significance (-Log_{10}(P-value) of the enrichment in each stage.

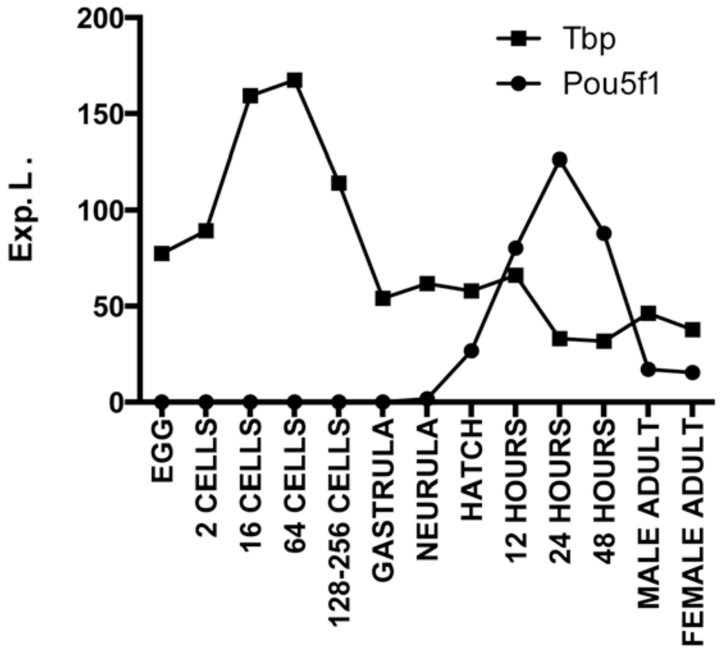

Supplementary Figure S3. Expression patterns of Pou5f1 and Tbp.

The expression levels of each gene at each stage were estimated by DGE-EM.

Supplementary Table S1. Summary of RNA-Seq raw data.

					RAW	TRIMMED	
Library	Sample	Technology	Insert size (bp)	Normalized	No. of Reads	No. of Reads	Throughput (bp)
Lib-1	adult of multiple individuals	454 SE	-	Yes	1,796,755	1,769,412	468,587,382
Lib-2	mixed embryos of different stages of multiple individuals	Solexa PE	200	Yes	25,540,743 * 2	16,237,278 * 2	2,215,273,942
Lib-3	mixed embryos of different stages of multiple individuals	Solexa PE	200	No	31,944,446 * 2	31,694,839 * 2	5,494,642,439
Lib-4 (SRR964432)	gill slits of multiple individuals	454 SE	-	-	476,739	471,527	156,031,127
Lib-5 (SRR964433)	gill slits of multiple individuals	454 SE	-	-	451,959	445,674	140,668,479
Lib-6 (SRR964434)	mixed embryos of different stages of multiple individuals	454 SE	-	-	594,167	588,521	208,864,399
Lib-7 (SRR964435)	mixed embryos of different stages of multiple individuals	454 SE	-	-	503,251	498,375	174,229,453
Lib-8 (SRR964436)	hepatic diverticulums and intestines of multiple individuals	454 SE	-	-	665,046	660,962	291,862,579
Lib-9 (SRR964438)	hepatic diverticulums and intestines of multiple individuals	454 SE	-	-	638,561	634,088	294,443,493
Lib-10 (SRR964444)	whole bodies of multiple individuals	Solexa PE	350	-	-	36,875,511 * 2	7,992,896,643
Lib-11 (SRR964474)	different embryonic stages of multiple individuals	Solexa PE	350	-	-	20,665,339 * 2	4,531,241,008
Lib-12 (SRR964578)	different embryonic stages of multiple individuals	Solexa PE	350	-	-	34,168,413 * 2	7,858,734,990
Lib-13 (SRR964579)	different embryonic stages of multiple individuals	Solexa PE	350	-	-	24,351,285 * 2	5,350,790,251
Lib-14 (SRR964580)	different embryonic stages of multiple individuals	Solexa PE	350	-	-	37,353,664 * 2	8,120,004,462
Lib-15 (SRR964581)	different embryonic stages of multiple individuals	Solexa PE	350	-	-	35,905,158 * 2	7,762,480,787
Lib-16 (SRR964582)	different embryonic stages of multiple individuals	Solexa PE	350	-	-	37,279,030 * 2	8,149,180,324
Lib-17 (SRR964583)	different embryonic stages of multiple individuals	Solexa PE	350	-	-	36,388,417 * 2	7,905,191,528

Supplementary Table S2. Summary of *de novo* assembly and merged transcript set.

Library	No. of transcripts	No. of mRNAs	CD-HIT-EST (RNA level)	CD-HIT (protein level)
Lib-1	142,972	41,075	15,266	17,304
Lib-2	84,211	29,925	16,846	14,791
Lib-3	212,139	63,474	35,673	32,696
Lib-3 + 2	247,087	74,001	39,642	35,428
Lib-3 + 2 + 1	247,798	74,453	39,598	35,373
Final merged (1-3)			61,492	48,400
Lib-4 + 5	84,314	22,021	10,362	11,967
Lib-6 + 7	13,809	4,327	2,923	3,279
Lib-8 + 9	58,788	34,646	12,461	13,895
Lib-10	362,898	101,913	54,537	46,946
Lib-11 - 17	1,753,405	142,880	75,980	63,392
Final merged			145,639	106,246

Supplementary Table S3. Assessment of completeness of transcripts by BLAST and CEGMA pipeline.

Library	Source [*1]	No. of existing *B. floridae* proteins [*2]	No. of existing CEGs	No. of complete CEGs
Lib-1	A	33,600	163	123 (49.60%)
	B	33,733	164	123 (49.60%)
	C	33,341	163	124 (50.00%)
Lib-2	A	35,708	191	129 (52.02%)
	B	35,834	191	129 (52.02%)
	C	35,673	191	129 (52.02%)
Lib-3	A	43,279	229	153 (61.69%)
	B	43,318	229	153 (61.69%)
	C	43,136	229	153 (61.69%)
Lib-3 + 2	A	44,388	228	154 (62.10%)
	B	44,434	227	154 (62.10%)
	C	44,177	229	155 (62.50%)
Lib-3 + 2 + 1	A	44,374	227	152 (61.29%)
	B	44,421	227	152 (61.29%)
	C	44,179	229	153 (61.69%)
Final merged (1-3)	A	NA	NA	NA
	B	45,055	243	213 (85.89%)
	C	44,887	244	216 (87.10%)
Lib-4 + 5	A	28,205	88	43 (17.34%)
	B	28,349	88	43 (17.34%)
	C	28,205	88	42 (16.94%)
Lib-6 + 7	A	15,634	43	19 (7.66%)
	B	15,699	43	19 (7.66%)
	C	15,106	43	19 (7.66%)
Lib-8 + 9	A	30,584	NA	NA
	B	30,371	NA	NA
	C	30,408	NA	NA
Lib-10	A	44,703	235	149 (60.08%)
	B	44,748	234	150 (60.48%)
	C	44,525	235	150 (60.48%)
Lib-11 - 17	A	46,498	238	193 (77.82%)
	B	46,526	239	193 (77.82%)
	C	45,879	239	193 (77.82%)
Final merged (all)	A	NA	NA	NA

	B#	47,159	247	240 (96.77%)
	C	46,948	245	237 (95.56%)
B. belcheri Annotation			NA	NA
B. floridae Annotation			242	224 (90.32%)

*1 Source: A. Original mRNA sets, B. Merged mRNA set by CD-HIT, C. Merged mRNA set by CD-HIT-EST.
*2 Search with 50,817 *B. floridae* proteins by BLASTp (E-value cutoff: 1E-10).
This transcript set with the best results when assessing the completeness by BLAST and CEGMA.

Supplementary Table S4. Summary of DGE-Seq data.

Library	Sample	No. of Reads	Throughput (Mbp)
a	Egg	2,399,202	40.79
b	2 Cells	2,643,806	44.94
c	16 Cells	2,804,807	47.68
d	64 Cells	4,977,234	84.61
e	128-256 Cells	2,624,557	44.62
f	Gastrula	3,341,922	56.81
g	Neurula	2,347,645	39.91
h	Hatch	3,099,621	52.69
i	12 Hours	2,934,949	49.89
j	24 Hours	2,966,370	50.43
k	48 Hours	3,162,146	53.76
l	Male Adult	3,817,146	64.89
m	Female Adult	4,402,578	74.84

Supplementary Dataset S1. List of stage-specific highly expressed transcripts.

See Supplementary Dataset S1.xls

Supplementary Dataset S2. List of Gene Ontology (GO) enrichment of each cluster.

See Supplementary Dataset S2.xls
